# Score‐based measurement invariance checks for Bayesian maximum‐a‐posteriori estimates in item response theory

**DOI:** 10.1111/bmsp.12275

**Published:** 2022-06-06

**Authors:** Rudolf Debelak, Samuel Pawel, Carolin Strobl, Edgar C. Merkle

**Affiliations:** ^1^ Department of Psychology University of Zurich Switzerland; ^2^ Epidemiology, Biostatistics and Prevention Institute (EBPI) University of Zurich Switzerland; ^3^ Department of Psychological Sciences University of Missouri Columbia MO USA

**Keywords:** item response theory, differential item functioning, Bayesian statistics

## Abstract

A family of score‐based tests has been proposed in recent years for assessing the invariance of model parameters in several models of item response theory (IRT). These tests were originally developed in a maximum likelihood framework. This study discusses analogous tests for Bayesian maximum‐a‐posteriori estimates and multiple‐group IRT models. We propose two families of statistical tests, which are based on an approximation using a pooled variance method, or on a simulation approach based on asymptotic results. The resulting tests were evaluated by a simulation study, which investigated their sensitivity against differential item functioning with respect to a categorical or continuous person covariate in the two‐ and three‐parametric logistic models. Whereas the method based on pooled variance was found to be useful in practice with maximum likelihood as well as maximum‐a‐posteriori estimates, the simulation‐based approach was found to require large sample sizes to lead to satisfactory results.

## 1 Introduction

Models of item response theory (IRT) are widely applied to describe the interaction of respondents and test items. A central question in empirical research is whether the estimated IRT parameters are invariant over the population of respondents. If this invariance assumption is found to be violated, this indicates that persons with the same ability differ with regard to their probability of correctly answering a specific item. In IRT, this type of model violation is typically related to differential item functioning (DIF; e.g., Holland & Wainer, 1993) and to the assessment of a test's fairness (Dorans & Cook, 2016).

Numerous methods have been proposed for the detection of violations of measurement invariance in the IRT framework. An important class of these methods investigates the invariance of parameters between predefined focal and reference groups (for an overview, see Magis, Béland, Tuerlinckx, & De Boeck, 2010). These groups are typically defined based on categorical person covariates (such as gender). If the methods are applied to detect DIF with respect to a continuous person covariate like age, it becomes necessary to categorize the person covariate. Previous studies have shown that an unfortunately chosen categorization may diminish these tests' sensitivity against DIF (e.g., Strobl, Kopf, & Zeileis, 2015).

Several approaches have recently been proposed to test the invariance of model parameters with regard to non‐categorical variables. These methods are based on different ideas and work quite differently from a statistical point of view, but all share the aim of allowing the inclusion of non‐categorical variables in invariance tests. A first approach sharing this aim is based on mixture distribution models. A well‐known example is the so‐called mixed Rasch model (Rost, 1990). A possible approach for using these models to check the stability of item parameters with regard to continuous covariates is based on a binary or multinomial logistic regression model. Here, the logistic regression model uses categorical or continuous covariates as predictors to model the mixing probabilities of latent classes for which the item parameters are allowed to differ (e.g., Dai, 2013; Li, Jiao, & Macready, 2016; Tay, Newman, & Vermunt, 2011).

A second approach that is not based on mixture distribution models aims to check the invariance of item response curves directly with regard to observed person covariates. Liu, Magnus, and Thissen (2016) presented a semiparametric approach that did not assume a specific parametrization of the item response curve and allowed the modelling of covariate effects on item responses. By defining a set of anchor items, whose item response functions are not moderated by the observed covariates, it is also possible to test for DIF in this framework. A similar model was proposed by Moustaki (2003).

A third approach is based on moderated nonlinear factor analysis (Bauer & Hussong, 2009; Bauer, 2017). Here, moderation functions are included in a factor model to specify an assumed relationship between its parameters (e.g., factor loadings) and observed person covariates. By estimating the parameters of these moderation functions (e.g., the slope parameters of linear functions), the invariance of the respective parameters of the factor model with regard to person covariates can be checked. A related method was recently described by Molenaar (2020).

Another approach, which is the focus of this paper, was recently proposed for a number of psychometric models and was motivated by methods for parameter invariance in econometrics (Andrews, 1993). These tests are based on the analysis of scores (i.e., the gradient of the model log‐likelihood with respect to the vector of the model parameters) and allow the detection of DIF effects with regard to a specific person covariate without the necessity to define focal and reference groups. Compared to the semiparametric approach of Liu *et al*. (2016), these score‐based tests are not based on the idea of incorporating the person covariate in the estimated model. Instead, they can be directly applied to models where person covariates were not considered in the parameter estimation. Moreover, they can be applied with categorical, ordinal and continuous covariates (Merkle & Zeileis, 2013; Merkle, Fan, & Zeileis, 2014).

This approach was applied to a wide range of psychometric models, including Bradley–Terry models (Strobl, Wickelmaier, & Zeileis, 2011), factor analysis (Merkle & Zeileis, 2013; Merkle *et al*., 2014), binary and polytomous Rasch models (Komboz, Strobl, & Zeileis, 2018; Strobl *et al*., 2015), normal‐ogive IRT models (Wang, Strobl, Zeileis, & Merkle, 2017), logistic IRT models (Debelak & Strobl, 2019) and mixed models (Fokkema, Smits, Zeileis, Hothorn, & Kelderman, 2018). The underlying theoretical foundation for these tests was provided by Zeileis and Hornik (2007), who investigated the problem of assessing the invariance of maximum likelihood (ML) estimators and M‐estimators. These tests are related to a family of score tests for detecting DIF with regard to a categorical covariate that was introduced by Glas and applied to numerous models (Glas, 1998, 1999, 2001, 2010a, 2010b; Glas & Suárez‐Falcón, 2003; Glas & van der Linden, 2010). A Bayesian variation of these tests for normal‐ogive IRT models, that is based on Lagrange multiplier tests, was described by Khalid and Glas (2016), and also aims to detect DIF effects with regard to a categorical covariate.

The first main contribution of this paper is the derivation and evaluation of two methods analogous to score‐based measurement invariance tests that are not based on the ML framework, but can also be applied to Bayesian estimators. Conceptually, the use of prior information allows the stabilization of item parameter estimates, and this paper addresses the extension of score‐based DIF tests to these estimators.

In applications of IRT, Bayesian maximum‐a‐posteriori (MAP) estimators were found to be more accurate than ML estimators, particularly in smaller samples. However, their application requires the definition of appropriate prior distributions. As Mislevy (1986) notes, poorly specified prior distributions might lead to a systematic bias in the estimation of groups of item parameters, which he named ‘ensemble bias’. These points were shown in several simulation studies, for example by Mislevy (1986), Harwell and Baker (1991) and Harwell and Janosky (1991). We follow those authors and others in essentially treating MAP estimates as potentially improved versions of ML estimates, applying hypothesis tests to the resulting estimates. Baker and Kim (2004) refer to this treatment as a ‘pragmatic’ use of Bayesian methodology, which can be contrasted with a fully Bayesian treatment that abandons null hypotheses and embraces posterior model uncertainty (see McElreath, 2015, for examples that specifically utilize MAP estimates). It is also possible to apply the ideas described here to the fully Bayesian treatment, a point on which we expand near the end of the paper.

We further consider multiple‐group IRT models, which allow the joint modelling of the interaction of test items with samples from multiple, heterogeneous populations and which can be used to account for ability differences between populations in DIF tests. As a second main contribution, we discuss an extension of the theoretical results of Zeileis and Hornik (2007), who essentially treat the single‐group case in the context of ML estimation, to these multiple‐group IRT models in the context of Bayesian MAP and ML estimation.

In the following sections we outline a statistical framework for score‐based model checks for MAP estimators and for multiple‐group IRT models. Based on this framework, we derive two families of approaches for score‐based DIF tests, a pooled variance approach and a simulation‐based approach. We compare these approaches for MAP and ML estimators by means of a simulation study, provide an empirical application, and discuss our findings in the final section. In the online supplementary materials we further present a brief tutorial for the application of both approaches in the R framework for statistical computing (R Core Team, 2020), which allows researchers to directly apply them to their own data.

## 2 Bayesian MAP item parameter estimation

Overviews on Bayesian IRT have been provided by Fox (2010), Levy and Mislevy (2016) and others. Here, we consider Bayesian MAP estimation (Baker & Kim, 2004; Mislevy, 1986), an estimation method that is implemented in several software packages, for instance the R package mirt (Chalmers, 2012). It is based on two principal ideas. The first idea is the definition of a distribution for the person parameters over which is integrated. This distribution is usually a normal distribution, although the parameters of this distribution (i.e., mean and variance) can differ for predefined person groups to account for impact effects (i.e., ability differences between the groups) (Bock & Zimowski, 1997). The second idea is the definition of a prior distribution for the item parameters. Bayesian MAP estimation can lead to more accurate item parameter estimates than frequentist estimation methods if the prior distributions are close to the true parameter distributions (Mislevy, 1986).

## 3 Score‐based DIF tests for ML and MAP estimators

In this section we summarize the principal ideas behind the score‐based DIF tests for ML estimators (e.g., Debelak & Strobl, 2019; Komboz *et al*., 2018; Merkle & Zeileis, 2013; Merkle *et al*., 2014; Strobl *et al*., 2011, 2015; Wang *et al*., 2017) to allow an assessment of the extent to which these tests can be applied to MAP estimators. We consider the score function, which is the vector of the first partial derivatives of the log‐likelihood with regard to the individual model parameters, that is, the gradient of the log‐likelihood. In the context of IRT models, these are typically the item parameters. In ML estimation, the parameters β are estimated so that this gradient is a null vector at the point of the estimator $\hat{\beta }$. In many IRT models the gradient is a sum over *N* individual score contributions $\psi \left(Y_{i},\hat{\beta }\right)$, where *Y*_*i*_ are the responses of person *i*. In summary, we obtain

(1) $$\sum_{i=1}^{N}\psi \left(Y_{i},\hat{\beta }\right)=0.$$

Instead of considering all *N* observations, we now consider the following stochastic process for $t\in \left[0,1\right]$ (⌊·⌋ denotes the floor function):

$$\bar{\Psi }\left(\hat{\beta },t\right)=\sum_{i=1}^{\left\lfloor \mathrm{Nt}\right\rfloor }\psi \left(Y_{i},\hat{\beta }\right).$$

From equation (1) it follows that $\bar{\Psi }\left(\hat{\beta },t\right)$ starts at 0 for *t* = 0 and also ends at 0 for *t* = 1. We now make the additional assumption that the individual score contributions are independent and identically distributed. This assumption typically holds, for instance, when marginal maximum likelihood (MML) estimation (Baker & Kim, 2004) is applied and a common distribution is assumed for all person parameters. It follows again from equation (1) that their expected value is 0 for all respondents. Let $\hat{V}$ be a consistent estimate of their covariance matrix. We now consider the following stochastic process for $t\in \left[0,1\right]$, which is a decorrelated cumulative sum process:

$$\Psi_{\mathrm{ML}}\left(\hat{\beta },t\right)={\hat{V}}^{-1/2}N^{-1/2}\sum_{i=1}^{\left\lfloor \mathrm{Nt}\right\rfloor }\psi \left(Y_{i},\hat{\beta }\right).$$

**Figure 1:**
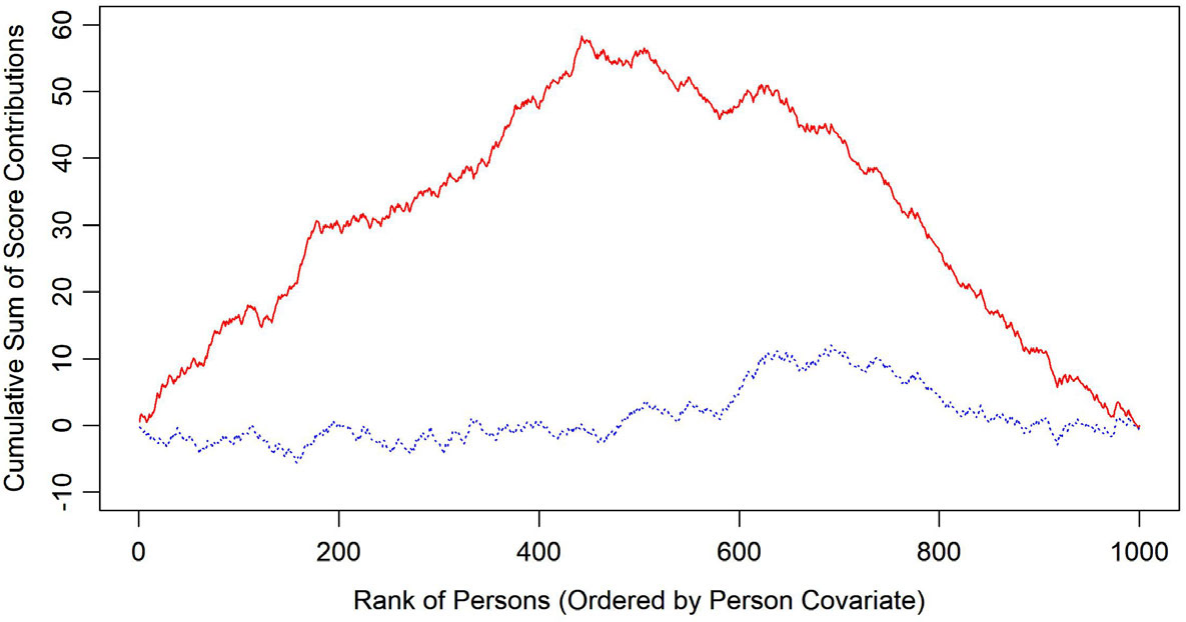
A typical example of cumulative score processes without (blue dotted line) and with (red solid line) DIF effect.

It can be shown that this process converges to a multidimensional standard Brownian bridge under the null hypothesis that DIF is absent (Zeileis & Hornik, 2007). It can be further shown that in the presence of DIF, the expected value of the individual score contributions is not 0 for individual respondents, but typically above 0 for some respondents and below 0 for others, depending on the direction of the DIF effect (*cf*. Figure 2 in Strobl *et al*., 2015). If the respondents are ordered with regard to a covariate that is related to DIF, the path of the cumulative sum process differs strongly from what is expected if DIF is absent. This idea is illustrated in Figure 1 using simulated data. As can be seen, the cumulative score process fluctuates randomly around 0 in the absence of DIF, whereas it deviates strongly from 0 when DIF is present. We observe that a centring of the individual score contributions does not affect this pattern.

In MAP estimation, the sum of the individual score contributions $\psi \left(Y_{i},\hat{\beta }\right)$, based on the model (i.e., item) parameter estimates $\hat{\beta }$, is equal to a term that we will label as *u*_prior_:

(2) $$\sum_{i=1}^{N}\psi \left(Y_{i},\hat{\beta }\right)=-u_{\text{prior}}\left(\hat{\beta }\right).$$

As our notation suggests, $u_{\text{prior}}\left(\beta \right)$ is the vector of the first derivatives of the logarithm of the prior distribution for β with respect to the individual estimated model (i.e., item) parameter; for a derivation of this result, see, for instance, Baker and Kim (2004, Section 7.4). This term depends on the prior distribution for β and on the item parameter estimate $\hat{\beta }$.

In contrast to the standard model used for ML estimators that was outlined above, the expected value of the individual score contributions is not 0, and the corresponding stochastic process is not a Brownian bridge. To obtain a similar standard model as in the case of ML estimation (Hjort & Koning, 2002; Zeileis & Hornik, 2007), it is thus necessary to centre the individual score contributions. In practical calculations, this can be achieved by simply subtracting their means, leading to centred individual score contributions $\tilde{\psi }\left(Y_{i},\hat{\beta }\right)$. This centring has the advantage that it shifts the expected value to 0, which corresponds to a shift in the values of all respondents to the same degree, but does not undo the effect of DIF, which corresponds to shifts in the values of different groups of respondents to different degrees. The pattern in the expected values of the individual score contributions, which occurs in the presence of DIF effects, is thus not affected by this overall centring of the individual score contributions.

It should be noted that, because of equation (2), $\tilde{\psi }\left(Y_{i},\hat{\beta }\right)$ converges to $\psi \left(Y_{i},\hat{\beta }\right)$ for increasingly large samples. This leads us to consider the following stochastic process, which corresponds to $\Psi_{\mathrm{ML}}$ for maximum likelihood estimates:

$$\Psi_{\mathrm{MAP}}\left(\hat{\beta },t\right)={\hat{V}}^{-1/2}N^{-1/2}\sum_{i=1}^{\left\lfloor \mathrm{Nt}\right\rfloor }\tilde{\psi }\left(Y_{i},\hat{\beta }\right).$$

As before, $\hat{V}$ is a consistent estimator of the covariance matrix of the individual score contributions. Using arguments similar to those used by Hjort and Koning (2002) and Zeileis and Hornik (2007), it can now be shown that this process converges to a standard Brownian bridge for N→∞. This result is a special case of Theorem 1, which will be presented below.

This asymptotic behaviour can be used for checking whether ML and MAP estimators in IRT are invariant with regard to a chosen person covariate if the individual score contributions are independent and identically distributed. In the next section we discuss a generalization of this approach to a more general scenario.

### 3.1 A generalization to multiple‐group IRT models

In this section we discuss score‐based tests of measurement invariance for models where the individual score contributions are independent, but not identically distributed. An important example are multiple‐group IRT models (Bock & Zimowski, 1997), where person parameter distributions are allowed to differ between groups of respondents that are defined before the analysis. These models thus allow the modelling of ability differences between known groups of respondents, which are also named impact effects, before the application of DIF tests. When considering score‐based tests for these models, one can therefore obtain individual score contributions for person group parameters. As we will show, score‐based tests can also be applied to MAP estimators in these models. In this extension, we assume that the prior distributions of the item parameters are independent of the underlying groups. In the DIF tests discussed in this paper, the score contributions from these group parameters were not further used in the calculation of the test statistics (see below). The underlying idea was to only use information from the item parameter estimates for the detection of DIF effects.

In this paper we focus on approaches that check the null model that all item parameters are invariant over all groups of respondents. This essentially leads to an overall DIF test for the item set. Since this test is applied to a model that includes impact effects, it can also discern between DIF and impact effects. Conceptually, it is also possible to adapt the test to define sets of anchor items and to check items or item groups for invariance. We will return to this point in Section 6. As will be shown, it is difficult to use a standard Brownian bridge as an approximation in this more general scenario. A possible approach for considering heterogeneity in the covariance matrices of the individual score contributions was discussed by Zeileis and Hornik (2007) for linear and generalized linear models. This first approach assumes that the covariance matrices stabilize with increasing sample size in a matrix $\hat{V}$, which can be used as an overall estimate of the covariance matrices in the population. In the context of multiple‐group IRT models, such an approach seems plausible, for instance, when there are many groups of different, but similar, ability.

In the context of multiple‐group IRT models, we will consider a second scenario, where there are few groups that differ strongly in their ability. In this scenario, the use of a common estimate $\hat{V}$ of the covariance matrix could lead to an overestimation of the variances of the individual score contributions in some ability groups and to an underestimation in others. This could further lead to a bias in the assumed distribution of the decorrelated individual score contributions under the null hypothesis of no DIF. Statistical tests based on such biased distributions can be expected to be either conservative or liberal.

To address this second scenario, we suggest using group‐specific covariance matrices for decorrelating the process. We now introduce the notation $V_{g\left(i\right)}$ to denote the covariance matrix of the score contributions in the group *g*(*i*), of which respondent *i* is a member. We further introduce ${\hat{V}}_{g\left(i\right)}$ to denote a consistent estimator for $V_{g\left(i\right)}$. Our main theoretical results for this scenario are summarized in the following theorem.

Theorem 1 Let $\tilde{\psi }\left(Y_{i},\hat{\beta }\right)$ denote the centred individual score contributions from a ML or MAP estimation. We consider the following stochastic process:

$${\tilde{\Psi }}_{\mathrm{MAP}}\left(\hat{\beta },t\right)=\frac{1}{\sqrt{N}}\sum_{i=1}^{\left\lfloor \mathrm{Nt}\right\rfloor }{\hat{V}}_{g\left(i\right)}{\left(\hat{\beta }\right)}^{-1/2}\tilde{\psi }\left(Y_{i},\hat{\beta }\right).$$

Since the individual score contributions $\tilde{\psi }\left(Y_{i},\hat{\beta }\right)$ are centred, they have to meet the following restriction:

$$\sum_{i=1}^{N}\tilde{\psi }\left(Y_{i},\hat{\beta }\right)=0.\;\left(*\right)$$

We can show the following characteristics for this process under the null hypothesis that DIF is absent and that the item parameter estimates are stable over the sample:

1. The expected values of the centred individual score contributions $E\left(\tilde{\psi }\left(Y_{i},\hat{\beta }\right)\right)$ are 0.
2. The expected values of the decorrelated individual score contributions $E\left({\hat{V}}_{g\left(i\right)}{\left(\hat{\beta }\right)}^{-1/2}\tilde{\psi }\left(Y_{i},\hat{\beta }\right)\right)$ are 0.
3. In both the single‐ and the multiple‐group case, ${\tilde{\Psi }}_{\mathrm{MAP}}\left(\hat{\beta },t\right)$ converges to a standard Brownian bridge under mild regularity conditions. In the single‐group case, its path ends at 0 for finite samples, whereas its path does generally not end at 0 for finite samples in the multiple‐group case.

The theoretical results summarized by this theorem are analogous to those reported by Zeileis and Hornik (2007), but concern a wider range of scenarios.^1^ Although the stochastic process ${\tilde{\Psi }}_{\mathrm{MAP}}\left(\hat{\beta },t\right)$ converges to a standard Brownian bridge, its path generally does not end at 0 for *t* = 1 in the multiple‐group case in finite samples, which is an important characteristic of Brownian bridges.

There seem to be two natural approaches for investigating this process in finite samples. First, we could use a single matrix for estimating the overall covariance matrix of the centred individual score contributions, which is similar to the single‐group case and leads to a path that ends at 0.

Second, we could aim to simulate the resulting stochastic process. Although there are several possible approaches, we propose the following solution: We generate data from a standard Brownian motion and then impose a restriction on it to make its path more similar to that of ${\tilde{\Psi }}_{\mathrm{MAP}}\left(\hat{\beta },t\right)$. This approach is inspired by a common method to generate data for a standard Brownian bridge, which consists of first generating data from a standard Brownian motion and then restricting its path to end at 0 for *t* = 1.

Below, we follow both approaches, with the first one leading to a pooled variance approach and the second one leading to a simulation‐based approach.

### 3.2 Summarizing empirical stochastic processes with test statistics

To compare the observed empirical stochastic process with that expected under the null model, it is necessary to define test statistics. Merkle and Zeileis (2013), Merkle *et al*. (2014) and Wang, Merkle, and Zeileis (2014) described several test statistics, which are also implemented in the software package strucchange (Zeileis, Leisch, Hornik, & Kleiber, 2002) of the statistical framework R (R Core Team, 2020). For brevity, we will focus on two test statistics which will be later used in our simulation studies. Several additional test statistics for continuous, ordinal and nominal covariates are available (Merkle *et al*., 2014; Wang *et al*., 2014).

Let $\gamma {\left(Y_{i},\hat{\beta }\right)}_{\mathrm{ij}}$ denote the centred and decorrelated individual score contributions of the empirical cumulative sum process, with *i* being an index for the *N* respondents and *j* being an index for the item parameters. For continuous covariates, we will use a *double max* statistic, which is given by (Merkle *et al*., 2014; Wang *et al*., 2014; Zeileis & Hornik, 2007)

$$\mathrm{DM}=\max_{i}\;\max_{j}|\gamma {\left(Y_{i},\hat{\beta }\right)}_{\mathrm{ij}}|.$$

For settings with a categorical covariate with *m* categories, we will use an *unordered Lagrange multiplier test*, which is given by (Merkle *et al*., 2014; Wang *et al*., 2014)

$${\mathrm{LM}}_{\mathrm{uo}}=\sum_{l}\sum_{j}{\left(\gamma {\left(Y_{i},\hat{\beta }\right)}_{i_{l}j}-\gamma (Y_{i},\hat{\beta }){}_{i_{l-1}j}\right)}^{2}.$$

In this equation, $i_{l}=\left\lfloor N\cdot t_{l}\right\rfloor$, where $t_{l}$, l=1,…,m−1, denotes the proportion of respondents in the first *l* categories.

### 3.3 Methods for calculating *p*‐values

As was outlined in the previous section, the stochastic processes based on the centred and decorrelated individual score contributions can be approximated by standard models in sufficiently large samples. However, the distribution of a test statistic for these stochastic processes remains unclear for finite samples. We will consider two approaches to determining the distribution of test statistics in multiple‐group IRT models, which conceptually correspond to the two approaches described in the previous subsection.

- *Pooled variance approach*. A first approach ignores possible differences in the groupwise covariances. Here, the centred individual score contributions are first calculated, and in a second step their common covariance matrix is calculated and used for decorrelating the score processes. The basic idea of this approach is to pool the covariance matrices to ensure that the decorrelated individual score contributions sum to 0, which in turn allows a standard Brownian bridge to be used as a reference model in finite samples. In the context of multiple‐group IRT models with a small number of groups, we consider this approach as a computational shortcut that may be useful in practical applications, particularly when impact effects are small and the groupwise covariance matrices can therefore be expected to be similar for all respondents. Computationally, it also allows a direct application of the framework of Zeileis and Hornik (2007) *via* the strucchange package in R (Zeileis *et al*., 2002). This approach has already been applied in previous studies with ML estimation in multiple‐group models (Debelak & Strobl, 2019; Wang *et al*., 2017), although not under this name.
- *Simulation‐based approach*. This approach corresponds to the approach based on groupwise covariance matrices. Here, we use simulated stochastic processes to obtain a reference distribution of the test statistic. To generate these stochastic processes, we use the fact that a Brownian motion, which we obtain as the asymptotic model in Theorem 1, also results when considering the cumulative sum of multidimensional standard normally distributed random variables. In this approach, the following steps are carried out to calculate *p*‐values:
  1. Calculate the empirical stochastic process ${\tilde{\Psi }}_{\mathrm{MAP}}\left(\hat{\beta },t\right)$.
  2. Obtain *N k*‐dimensional draws from a multivariate standard normal distribution, with *N being* our empirical sample size and *k* being the number of estimated item parameters. Each draw corresponds to an empirical observation. Since these draws are normally distributed, their cumulative sum process can be used to simulate a Brownian motion.
  3. Apply a groupwise decorrelation and calculate cumulative sums to obtain a multivariate stochastic process. Apply a linear transformation to these draws so that their sum matches ${\tilde{\Psi }}_{\mathrm{MAP}}\left(\hat{\beta },1\right)$, that is, the end point of the path of ${\tilde{\Psi }}_{\mathrm{MAP}}$. The path of this process now has the same start and end point as ${\tilde{\Psi }}_{\mathrm{MAP}}\left(\hat{\beta },t\right)$.
  4. Repeat steps 2 and 3 many (e.g., 1,000) times and calculate a suitable test statistic for every simulated path. This leads to a reference distribution of test statistics.
  5. Calculate *p*‐values by comparing the observed value of the test statistic with the reference distribution calculated in step 4.

This approach was also inspired by Zeileis and Hornik (2007), who also mentioned the simulation of the asymptotic model as a method for determining *p*‐values. We emphasize that the motivation for the linear transformation in step 3 is to make the observed and the simulated processes more similar under the null hypothesis that no DIF is present so that DIF effects can be detected more easily. Other approaches for simulating ${\tilde{\Psi }}_{\mathrm{MAP}}\left(\hat{\beta },t\right)$ could be based on bootstrapping, or make specific use of the group membership of the respondents while simulating the process. A systematic investigation of various variations of this algorithm is, however, beyond the scope of this paper.

Since this method is based on asymptotic results, it can be expected to lead to accurate results in sufficiently large samples. However, its applicability in finite samples can also be expected to depend on the rate of convergence to this asymptotic model, which in turn can be expected to depend on the distribution of the individual score contributions. For instance, a sum of only a few individual score contributions with a very skewed distribution cannot be expected to be described well by a sum of normally distributed random variables in small samples. For larger samples, however, this can be expected because of the central limit theorem.

So far, we have discussed these approaches for a scenario with multiple groups where a groupwise decorrelation can be applied. Both approaches can also be straightforwardly applied in single‐group IRT models. In summary, the proposed model checks consist of the following steps: First, item parameter estimates are calculated. Second, individual score contributions are calculated as a measure of personwise model fit. If MAP estimation is used, the individual score contributions are centred to take the prior distributions into account. The score contributions should fluctuate randomly around 0 if the parameters are invariant. Third, decorrelation is applied to obtain a standardized stochastic process. Fourth, a suitable test statistic is chosen. In a final step, one of the outlined methods is applied to calculate *p*‐values.

The proposed procedures are based on large‐sample arguments and it is unclear to what extent they are applicable in finite samples. In the next section, we report a simulation study that investigates these model checks for finite samples under conditions with and without parameter invariance.

## 4 An evaluation with a simulation study

To evaluate the proposed method, we evaluate the rate of extreme *p*‐values of the resulting model checks in a simulation study. Since the original ML approach can be considered as a special case that should correspond to the use of a non‐informative prior, the tests for ML estimators are included to serve as a reference method. This simulation study considers scenarios where measurement invariance with regard to a categorical or continuous person covariate is investigated.

### 4.1 Method

We first describe the data‐generating processes in this simulation study before proceeding to the data analysis. The data sets generated differed with regard to the following conditions:

- *Type of IRT model*. The data sets were generated and analysed based on the two‐parametric logistic (2PL) or three‐parametric logistic (3PL) model (Birnbaum, 1968). Bayesian MAP estimators are sometimes recommended for the 3PL model to obtain more accurate item parameter estimates (e.g., Mislevy, 1986). We used the following response function to generate data under the 3PL model:
$$P\left(Y_{\mathrm{ij}}=1¦a_{j},d_{j},c_{j},\theta_{i}\right)=\left(c_{j}+\Delta c_{j}\right)+\frac{1-\left(c_{j}+\Delta c_{j}\right)}{1+\exp\left(-\left(a_{j}+\Delta a_{j}\right)\cdot \theta_{i}-\left(d_{j}+\Delta d_{j}\right)\right)}.$$
  Here, *Y*_*ij*_ corresponds to the response of the *i*th respondent to the *j*th item, with 1 denoting a positive and 0 a negative response. θ_*i*_ is an ability parameter for respondent *i*. *a*_*j*_, *d*_*j*_ and *c*_*j*_ are item parameters, with *a*_*j*_ being a slope and *d*_*j*_ an intercept parameter for the item response function. *c*_*j*_ is a pseudo‐guessing parameter. The terms Δ*a*_*j*_, Δ*d*_*j*_ and Δ*c*_*j*_ correspond to DIF effects. When the 2PL model was used, *c*_*j*_ and Δ*c*_*j*_ were set to 0.
- *Number of respondents*. The simulated data sets consist of either 500, 1,000, 2000 or 5,000 respondents. These numbers correspond to those typically found in psychological and educational studies, where the 2PL and 3PL models could be applied.
- *Number of items and item parameters*. The simulated item sets had a size of 10 or 30 items. The slope parameters were sampled from a log‐normal distribution $LN\left(0,0.0625\right)$. The intercept parameters were sampled from a normal distribution $N\left(0,1\right)$. Under conditions with the 3PL model pseudo‐guessing parameters were sampled from a beta distribution $B\left(5,45\right)$. These distributions were inspired by prior distributions proposed in the literature (Culpepper, 2016; Fox, 2010).
- *Sampling of the person covariate and presence of an impact effect*. The simulated person covariate could be either categorical or continuous. Under conditions with a categorical covariate, this covariate was determined by randomly assigning each respondent to one of two groups, with each respondent having a probability of.5 of being assigned to the first group. The covariate was subsequently used to simulate DIF and impact effects. If an impact effect was present, the person parameter distribution for all respondents in the first group was $N\left(-0.5,1\right)$, whereas it was $N\left(0.5,1\right)$ for all respondents in the second group. Under conditions with a continuous covariate, the person covariate was sampled from a uniform distribution with a minimum of 20 and a maximum of 70. This distribution aimed to resemble a person covariate like age. The conditional distribution of the person ability parameters was standard normally distributed for all values of this covariate. Based on this covariate, conditions with and without impact effects were simulated. If an impact effect was present, the person parameter distribution for all respondents with a covariate value below 35 was $N\left(-0.5,1\right)$, and for all respondents with a covariate value of 35 or above was $N\left(0.5,1\right)$. This condition aimed to simulate a cohort effect. If no impact effect was simulated, all person parameters were drawn from a standard normal distribution $N\left(0,1\right)$.
- *Presence and type of DIF effect*. In addition to a baseline condition where no DIF effects were present, several conditions with DIF were included. Under conditions with a continuous covariate, DIF effects led to differences in the item parameters between respondents with a covariate value below 35 and those with a covariate value of 35 or higher. Under conditions with a categorical covariate, DIF effects were simulated between the two respondent groups. The conditions with DIF effects varied with regard to the following two points:
  - *Parameters affected by DIF*. DIF was simulated for either the intercept, the slope, or, when the 3PL model was used, the pseudo‐guessing parameter. For one‐fifth of the item set, DIF was simulated in the selected parameter. The items affected by DIF were randomly selected.
  - *Size and direction of the DIF effects*. The parameter change between the two groups was set to $\Delta a_{j}=0.3$ for the slope parameter, $\Delta d_{j}=0.6$ for the intercept parameter, and $\Delta c_{j}=0.1$ for the pseudo‐guessing parameter. There were two conditions on the direction of the DIF effect. Under a balanced DIF effect, the item parameters increased for one half of the items affected by DIF, whereas they decreased for the other half. Under a condition with an unbalanced DIF effect, the item parameters increased for all items affected by DIF. If an impact effect was presented, this increase favoured the more able group. Again, these numbers were inspired by previous studies (e.g., Debelak & Strobl, 2019).
- *Estimation method*. The model parameters were estimated by MML or MAP. In both cases an impact effect was modelled by a multiple‐group IRT model (Baker & Kim, 2004) that constrained all item parameters to be invariant for the whole sample. In the item parameter estimation, this effect was considered by assuming a standard normal distribution for the ability parameters of the first of these groups and a normal distribution $N\left(\mu ,\sigma^{2}\right)$ for the ability parameters of the second group. μ and σ^2^ were estimated from the data using an ML estimator. After estimating the model parameters, score‐based tests were conducted based on a double maximum statistic when the person covariate was continuous and based on an unordered Lagrange multiplier statistic when the person covariate was categorical. This corresponds to the recommendations made by Merkle and Zeileis (2013), Merkle *et al*. (2014) and Wang *et al*. (2014).
  The application of a Bayesian MAP estimator made the definition of prior distributions for all item parameters necessary. We used two sets of prior distributions:
  - *Agreeing prior*. This prior aimed to depict a scenario where information about the distribution of item parameters is available. In conditions with the 3PL model, the prior distribution of the pseudo‐guessing parameter was set to $B\left(4,45\right)$, corresponding to the distribution from which these parameters were drawn. For the slope and intercept parameters, the prior distributions also corresponded to the distributions from which these parameters were drawn. The prior distribution for the slope parameters was thus $LN\left(0,0.0625\right)$, whereas it was $N\left(0,1\right)$ for the intercept parameters. These priors were used independently of whether these item parameters were estimated for the 2PL or the 3PL model.
  - *Non‐informative prior*. Here, we used non‐informative prior distributions for all parameters. For the slope parameters, a normal distribution $N\left(1,10\right)$ was used as prior distribution, whereas $N\left(0,10\right)$ was used for the intercept parameters. This pertained to all conditions. Under conditions with the 3PL model, B(1,1) was used as prior for the pseudo‐guessing parameter.

Under each condition, 1,000 data sets were generated. In each data set, first the item parameters were estimated using the estimation methods presented. As a second step, parameter invariance was investigated for all parameter estimates with the pooled variance approach for the first 500 data sets and the simulation‐based approach for the remaining 500 data sets. In the simulation‐based approach, 1,000 artificial data sets were generated per observed data set to obtain a reference distribution for the calculation of *p*‐values. We investigated the rate of extreme *p*‐values below .05 for each condition and each method.

### 4.2 Results under parameter invariance

We first present results on the Type I error rates. Overall, the Type I error rate was found to depend strongly not only on the testing approach used, but also on the underlying IRT model, the sample size and the form of the prior distribution. This was overall not surprising, since these factors affect how well the observed cumulative sum processes can be approximated by Brownian bridges. In particular, we found the individual score contributions of the pseudo‐guessing parameter to be skewed, which generally led to an increased Type I error rate in the 3PL model in smaller samples.

#### 4.2.1 Results for a categorical covariate

**Figure 2:**
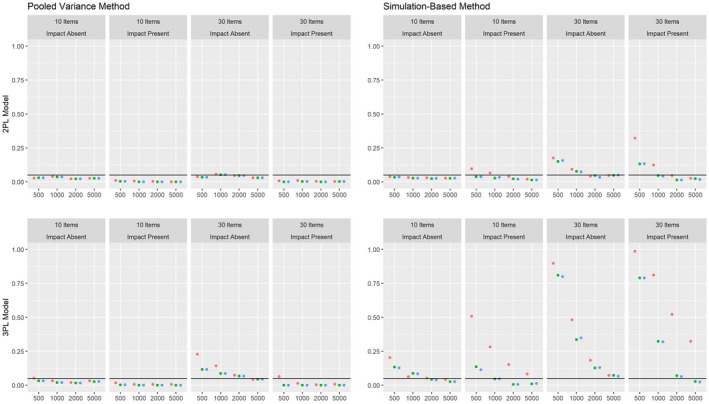
Type I error rate for various conditions of test length, sample size and presence of impact with a categorical covariate for MML estimation (green) and MAP estimation with a non‐informative (blue) and an agreeing prior (red).

The results on the Type I error rates for conditions with a categorical covariate are summarized in Figure 2. Overall, the pooled variance approach leads to a rather conservative test under these conditions, with the only exception being the 3PL model applied to samples of 500 or 1,000 respondents and 30 items.

Overall, the simulation‐based approach leads to satisfying results for the 2PL model under conditions with a sample size of 1,000 respondents or more. For the 3PL model, the Type I error rate was strongly increased for conditions with 30 items, but was close to the nominal alpha level of .05 for conditions with 10 items and samples with 1,000 respondents or more. For both approaches, MAP estimation with a non‐informative prior and ML estimation led to comparable results overall, while the Type I error rate was increased for MAP estimation with an agreeing prior under some conditions. For the pooled variance approach, the Type I error rate was slightly lower for the conditions with impact effects than for the conditions without impact effects. The simulation‐based approach, on the other hand, showed an overall comparable Type I error rate for conditions with and without impact, with the only exception being conditions where an agreeing prior was used.

#### 4.2.2 Results for a continuous covariate

**Figure 3:**
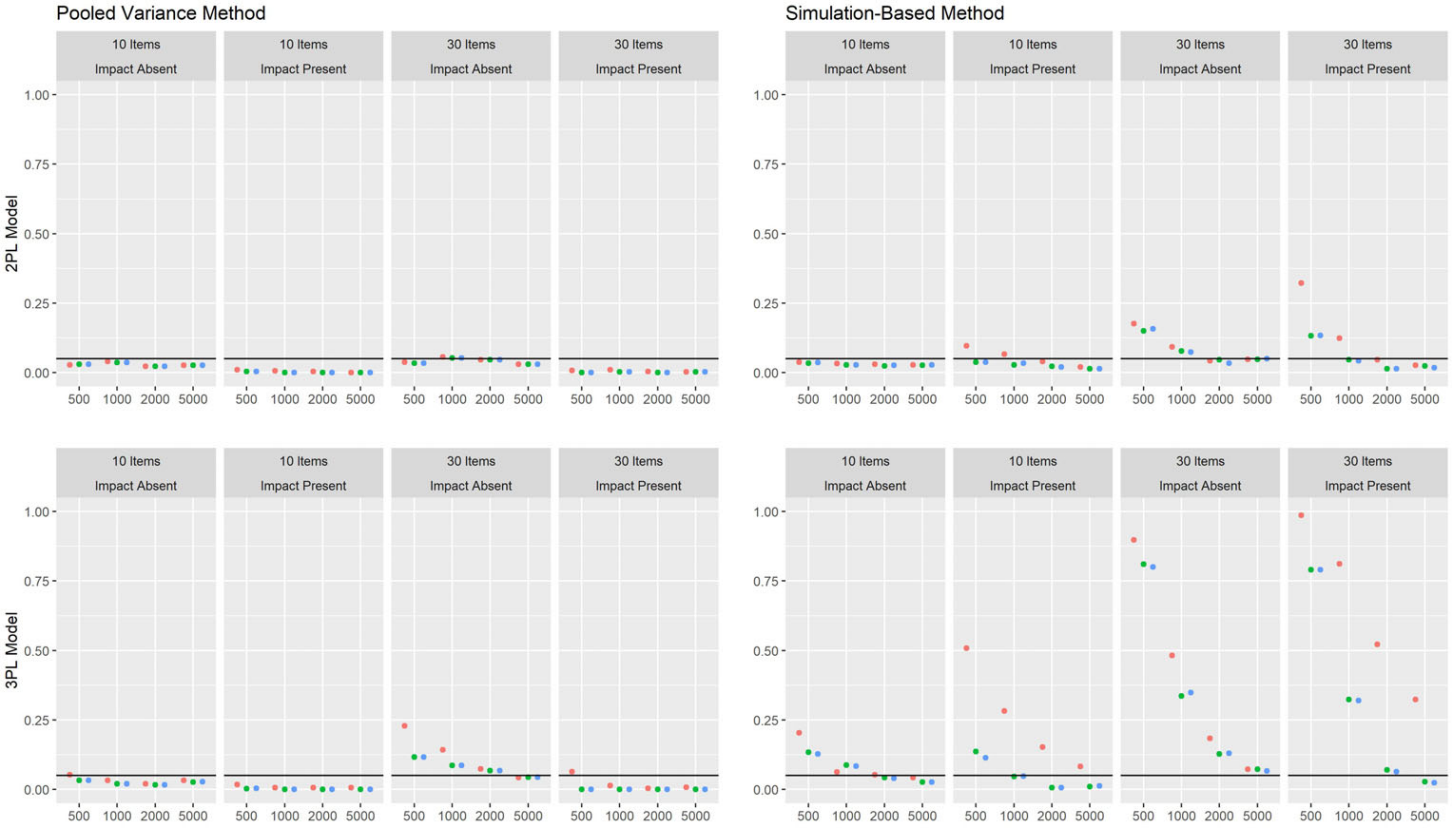
Type I error rate for various conditions of test length, sample size and presence of impact with a continuous covariate for MML estimation (green) and MAP estimation with a non‐informative (blue) and an agreeing prior (red).

The results on the Type I error rate for conditions with a continuous covariate are summarized in Figure 3. Here, the pooled variance approach leads to satisfying results under all conditions. The simulation‐based approach shows Type I error rates close to the nominal alpha level for the 2PL model for samples of about 1,000 or more respondents. For the 3PL model, the Type I error rate was strongly increased, with the only exceptions being some conditions with very large samples. Again, MAP estimation with a non‐informative prior and ML estimation led to overall comparable results, while the Type I error rate was increased for MAP estimation with an agreeing prior under some conditions. Whereas conditions with and without impact effects led to comparable results for the pooled variance method, the simulation‐based method showed an overall increased Type I rate when impact was present.

### 4.3 Results on the sensitivity against DIF effects in the 2PL model

#### 4.3.1 Results for a categorical covariate

**Figure 4:**
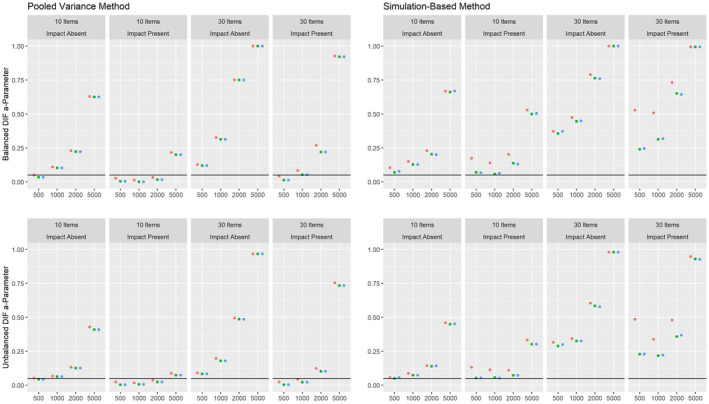
Power against DIF in the slope parameter in the 2PL model for various conditions of test length, sample size and presence of impact with a categorical covariate for MML estimation (green) and MAP estimation with a non‐informative (blue) and an agreeing prior (red).

**Figure 5:**
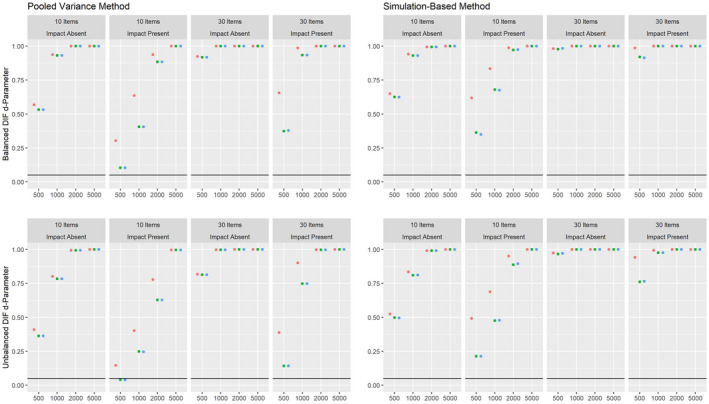
Power against DIF in the intercept parameter in the 2PL model for various conditions of test length, sample size and presence of impact with a categorical covariate for MML estimation (green) and MAP estimation with a non‐informative (blue) and an agreeing prior (red).

Figures 4 and 5 present the results on power against DIF in the slope parameter and intercept parameter, respectively, for the 2PL model for conditions with a categorical covariate. As can be seen, both the pooled variance and the simulation‐based approach have power against the simulated DIF effects. Overall, the simulation‐based approach had slightly more power, but also showed an increased Type I error rate under conditions with 500 respondents and 30 items. For both approaches, the power was overall higher in conditions where impact was absent.

#### 4.3.2 Results for a continuous covariate

**Figure 6:**
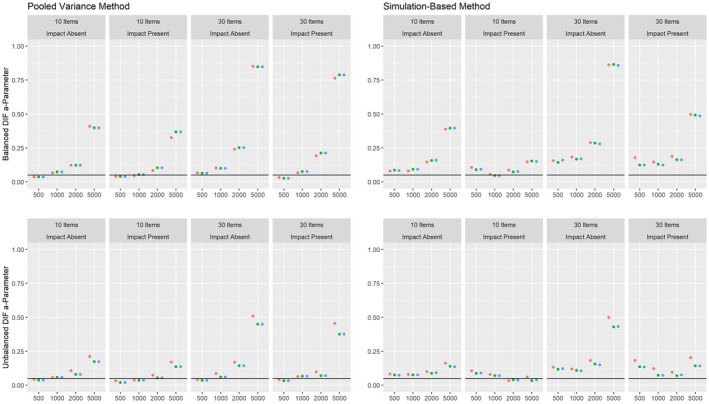
Power against DIF in the slope parameter in the 2PL model for various conditions of test length, sample size and presence of impact with a continuous covariate for MML estimation (green) and MAP estimation with a non‐informative (blue) and an agreeing prior (red).

**Figure 7:**
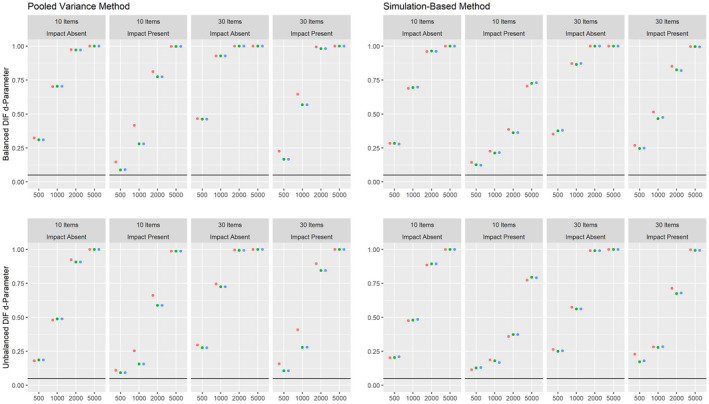
Power against DIF in the intercept parameter in the 2PL model for various conditions of test length, sample size and presence of impact with a continuous covariate for MML estimation (green) and MAP estimation with a non‐informative (blue) and an agreeing prior (red).

Figures 6 and 7 present the corresponding results on power against DIF in the slope parameter and intercept parameter, respectively, for conditions with a continuous covariate in the 2PL model. Again, both approaches showed power against DIF effects, although the pooled variance approach had more power under these conditions. Again, the power was higher overall in conditions where impact was absent for both approaches.

### 4.4 Results on the sensitivity against DIF effects in the 3PL model

#### 4.4.1 Results for a categorical covariate

**Figure 8:**
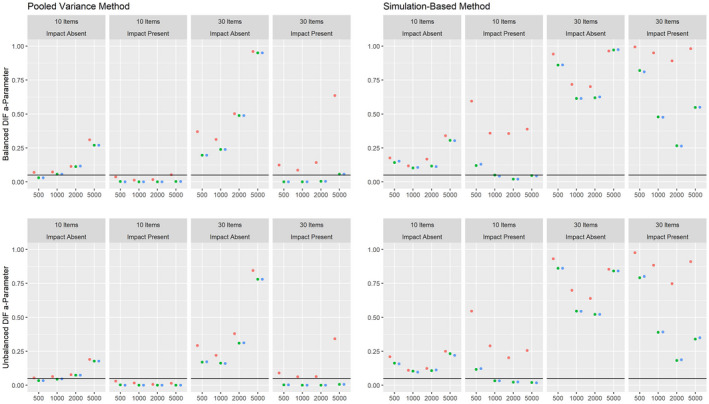
Power against DIF in the slope parameter in the 3PL model for various conditions of test length, sample size and presence of impact with a categorical covariate for MML estimation (green) and MAP estimation with a non‐informative (blue) and an agreeing prior (red).

**Figure 9:**
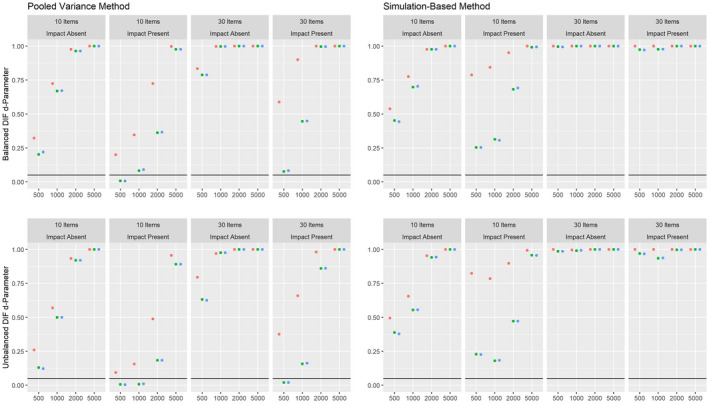
Power against DIF in the intercept parameter in the 3PL model for various conditions of test length, sample size and presence of impact with a categorical covariate for MML estimation (green) and MAP estimation with a non‐informative (blue) and an agreeing prior (red).

**Figure 10:**
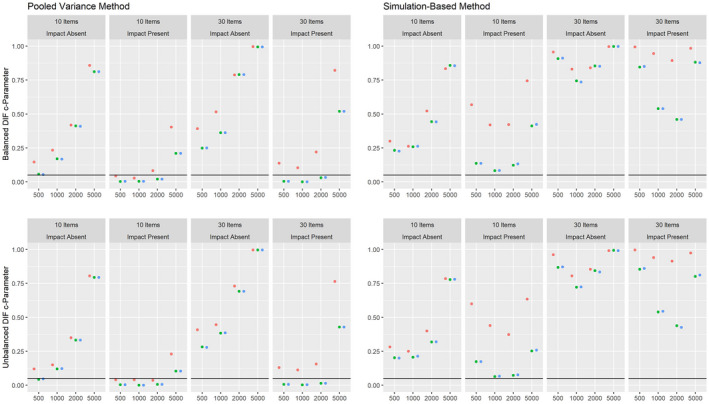
Power against DIF in the pseudo‐guessing parameter in the 3PL model for various conditions of test length, sample size and presence of impact with a categorical covariate for MML estimation (green) and MAP estimation with a non‐informative (blue) and an agreeing prior (red).

Figures 8, 9, 10 present the results on power against DIF in the slope, intercept and pseudo‐guessing parameter for the 3PL model for conditions with a categorical covariate. Overall, the results mirror those for the 2PL model, although it should be considered that the simulation‐based approach showed an increased Type I error rate for conditions with 30 items for the 3PL model. The high rate of positive rates in small samples should therefore not be interpreted as an indicator of high power, but indicate that the method tends to provide positive results in small samples for the 3PL model. Both approaches showed only low power against DIF in the slope parameter. As was the case for the 2PL model, the power was higher overall in conditions where impact was absent.

#### 4.4.2 Results for a continuous covariate

**Figure 11:**
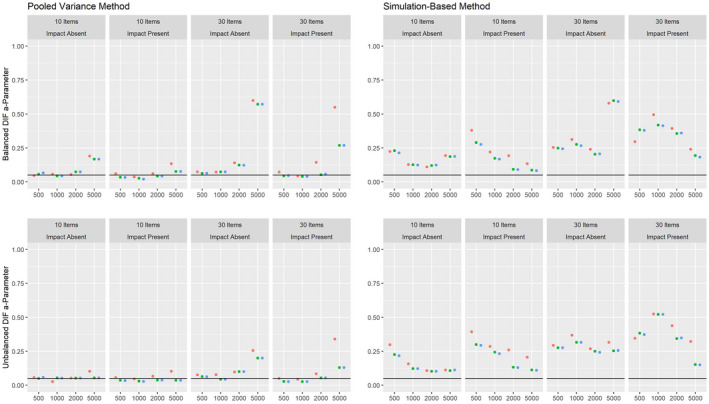
Power against DIF in the slope parameter in the 3PL model for various conditions of test length, sample size and presence of impact with a continuous covariate for MML estimation (green) and MAP estimation with a non‐informative (blue) and an agreeing prior (red).

**Figure 12:**
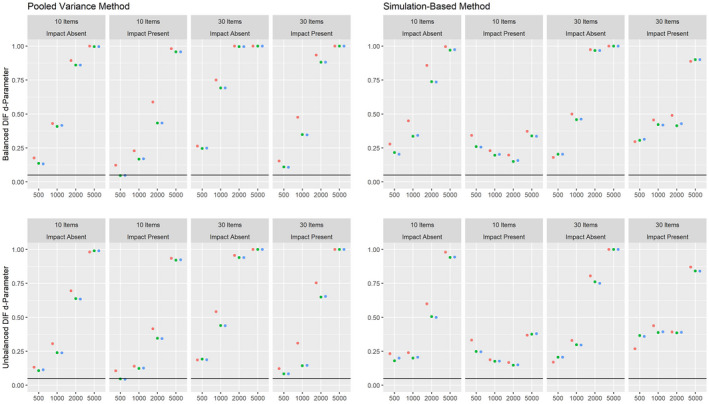
Power against DIF in the intercept parameter in the 3PL model for various conditions of test length, sample size and presence of impact with a continuous covariate for MML estimation (green) and MAP estimation with a non‐informative (blue) and an agreeing prior (red).

**Figure 13:**
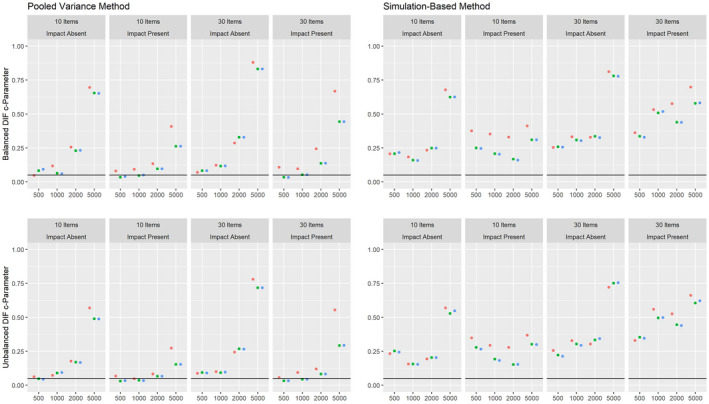
Power against DIF in the pseudo‐guessing parameter in the 3PL model for various conditions of test length, sample size and presence of impact with a continuous covariate for MML estimation (green) and MAP estimation with a non‐informative (blue) and an agreeing prior (red).

Figures 11, 12, 13 present the corresponding results on power against DIF in the slope, intercept and pseudo‐guessing parameter for conditions with a continuous covariate. Here, we focus on the results for the pooled variance approach, since the simulation‐based approach showed an increased Type I error rate. It can be seen that this approach has power against all simulated DIF effects, given a sufficiently large sample. Again, we found higher power in conditions without impact effects.

## 5 An empirical application

As an illustration of the new method, we demonstrate its application to the MathExam14W data set from the psychotools package (Zeileis, Strobl, Wickelmaier, Komboz, & Kopf, 2020). This data set contains the responses of 729 business and economics students to 13 items in a written introductory mathematics exam at the University of Innsbruck, Austria. The items were single‐choice items with five response options per item, and aimed to assess the basics of analysis, linear algebra and financial mathematics. Eight items (1, 5, 6, 7, 8, 9, 11, 12) were used in two different versions in this exam. The two versions differed in their wording, but aimed to assess the same skills; moreover, one version was presented in the morning, whereas the other one was presented later, after the assessment of the first group had finished. We want to investigate parameter invariance for these two groups of respondents.

In this illustration we are modelling the observed responses with the 3PL model, which seems plausible for this data set. We are further interested whether the item parameters differ between the two groups. The R code for reproducing this demonstration is available in the Supporting Information. In this application, we are comparing two estimation methods. The first method is a score‐based DIF test where all item parameters are estimated by an MML approach. The second method employs an MAP approach, using a non‐informative prior. This estimation method uses flat normal distributions centred around 0 and 1 for the intercept and slope parameters, respectively. The non‐informative prior uses a uniform distribution between 0 and 1 for the guessing parameters. We first present the estimated parameter values under the two approaches in Table 1.

**Table 1:** Estimated slope (*a*), intercept (*d*) and pseudo‐guessing (*c*) item parameters using MML and MAP estimation

Item number	*a* (MML)	*a* (MAP)	*d* (MML)	*d* (MAP)	*c* (MML)	*c* (MAP)
1	3.797	3.778	−3.323	−3.249	0.423	0.421
2	1.479	1.463	1.111	1.120	0.000	0.000
3	1.532	1.529	1.411	1.423	0.000	0.000
4	1.859	1.855	−0.712	−0.689	0.183	0.181
5	1.244	1.240	1.054	1.063	0.000	0.000
6	1.501	1.492	0.785	0.796	0.000	0.000
7	23.191	6.389	−27.543	−7.708	0.106	0.099
8	2.126	2.123	0.851	0.868	0.000	0.000
9	27.480	6.150	−21.096	−4.627	0.285	0.272
10	2.381	2.507	−1.334	−1.407	0.138	0.145
11	2.293	2.258	1.960	1.965	0.000	0.000
12	1.793	1.790	0.756	0.770	0.000	0.000
13	1.271	1.353	−0.990	−1.067	0.151	0.164

As can be seen, the MML and MAP estimations tend to lead to numerically comparable item parameter estimates for most items. For items 7 and 9, the ML approach leads to extreme values for the slope and intercept parameters, which are not observed under the non‐informative prior. The *p*‐values for both estimation methods are close to 0 using the simulation‐based and pooled variance approaches, which indicates a violation of measurement invariance for at least one item parameter. It should be noted that the pooled variance approach was found to be conservative in the simulation studies under similar conditions, whereas the simulation‐based approach was found to be slightly liberal (see the bottom left panels for the pooled variance and simulation‐based approach in Figure 2).

## 6 Discussion

This study has discussed the application of theoretical results analogous to those of Zeileis and Hornik (2007) and Hjort and Koning (2002) for testing the invariance of model parameters in the context of Bayesian MAP estimation and multiple‐group IRT models. We argued that this framework can still be applied after a centring and a groupwise decorrelation of the individual score contributions, and we discussed standard models which could be used for testing the invariance in sufficiently large samples. Our considerations led to two alternative approaches: a pooled variance approach and a simulation‐based approach. The approaches presented lead to DIF tests that check the null model that all item parameters in an item set are invariant with regard to a categorical or continuous person covariate.

The pooled variance approach is based on the assumption that the covariance matrices of the individual score contributions can be treated as equal. If this assumption is met, *p*‐values are calculated *via* an asymptotic model. Under the conditions used in our simulation study, this approach was found to be conservative, and could still be applied in rather small samples. It was also found to be sensitive against various forms of DIF effects, although its power also depended on the sample size, test length and type of DIF effect. Future work should investigate possible practical limitations of this approach that could occur if the underlying assumption of equal covariance matrices is strongly violated.

The simulation‐based approach, on the other hand, aims to simulate data from a reference model that should asymptotically hold if the parameters are invariant. As the original approach of Zeileis and Hornik (2007), this approach is based on a functional limit theorem and therefore requires sufficiently large samples. This approach tended to show an increased Type I error rate in small samples. Conceptually, this approach relies on assumed approximation of the cumulative score processes of all item parameters by a restricted standard Brownian motion. If the number of items increases, this approximation is more likely not to hold for individual items. It follows that this approach can be expected to become more unreliable with an increasing number of items, which corresponds to the results of our simulation study. Furthermore, this approximation can be expected to become more unreliable when the individual score contributions show a distribution with a high skewness. In our simulation studies, this was observed for the individual score contributions of pseudo‐guessing parameters. This observation might explain why the Type I error rate of the simulation‐based approach was generally much higher for the 3PL model than for the 2PL model.

Our results still indicate that this approach can be useful in the comparatively simple and widely used 2PL model and, for large samples and short tests, the 3PL model. Under several conditions, the power of this approach exceeded that of the pooled variance approach.

An important characteristic of both approaches is that, since they depend on asymptotic results for the cumulative sums of the individual score contributions, their power and Type I error depend on the chosen prior distribution. This is illustrated by the results of the simulation study.

Additional testing approaches can be imagined in the score‐based framework presented. A first extension, already discussed in the previous literature (Merkle & Zeileis, 2013; Merkle *et al*., 2014), pertains to the use of additional test statistics. Second, one could use additional methods for the calculation of *p*‐values besides the pooled variance and simulated‐based approaches presented. For instance, one could use approaches based on the resampling of the individual score contributions (e.g., permutation or bootstrap tests) to detect significant deviations from the null model. In a small simulation study, we found that such tests did not offer practical advantages over the pooled variance and simulation‐based methods, but future work could investigate such tests for several alternative conditions and IRT models.

A potential limitation of the evaluated approaches is that we focused on multiple‐group IRT models where all items were restricted to be invariant to estimate impact effects. This restriction is related to the tested null model, which also assumes that all item parameters are invariant, and it might not be suitable for applications where some items cannot be considered to be stable beforehand. As a possible solution, one could further adapt the presented procedure to obtain an itemwise DIF test for individual items or item groups which assumes the invariance of a selected set of anchor items. This set of anchor items would also be used for estimating impact effects. These tests would differ in two aspects from the tests presented here. First, an IRT model would be estimated where the item parameters of both the anchor items and the items that will be investigated for DIF are restricted to be invariant; item parameters of any other items would be allowed to differ between groups of respondents. Second, the test statistics of the score‐based tests would be calculated using only processes that correspond to item parameters of items that we want to test for DIF. The overall DIF tests evaluated in this paper result as a special case, where all items are simultaneously tested for parameter invariance. The evaluation of these itemwise DIF tests is left as a topic for future work.

Another interesting question concerns the robustness of this approach against violations of the assumptions made in the impact and item parameter estimation, such as a misspecification of the groups used and the distribution of the ability parameters. Debelak and Strobl (2019) investigated a similar research question for the pooled variance approach in two simulation studies and found it to be robust against moderate violations of the underlying assumptions. However, their study also found an increased Type I error rate of this test when an impact effect related to the covariate tested for DIF is not modelled. Future research might further investigate the robustness of the proposed methods.

Finally, similar approaches could be applied in a full Bayesian approach. A possible and straightforward extension of this kind could determine posterior predictive *p*‐values for Bayesian model checks. These possible extensions should be investigated and evaluated in future work.

## 7 Computational details

All calculations were carried out in the R framework for statistical computing (R Core Team, 2020), version 4.0.2. The MAP and MML estimators were calculated using the mirt package (Chalmers, 2012), version 1.30. The pooled variance approach was calculated with the strucchange package (Zeileis *et al*., 2002), version 1.5–1. The simulation‐based approach was applied with R code that was written specifically for this study.

## Conflicts of interest

All authors declare no conflict of interest.

## Author contributions

**Rudolf Debelak:** Conceptualization; investigation; methodology. **Samuel Pawel:** Software. **Carolin Strobl:** Resources. **Edgar C. Merkle:** Conceptualization; methodology.

## Supporting information


**Appendix S1.** Application in R.

## Footnotes

^1^
Although we do not present a proof here, a draft of the proof of Theorem 1 can be obtained from the first author.
