# Supplementary material for: Score‐based measurement invariance checks for Bayesian maximum‐a‐posteriori estimates in item response theory
Source: Br J Math Stat Psychol. 2022 Jun 6;75(3):728–52. doi: 10.1111/bmsp.12275 (PMC9796736; doi:10.1111/bmsp.12275)
Supplement: Supplementary file 1 — Appendix S1. Application in R. [file BMSP-75-728-s001.pdf]

Supplementary Material: Application in R

In this supplementary document, we outline the application of both the pooled variance and the simulation-based approach using published R packages. In a first section, we describe some R functions that are necessary for running the tests before showing their application in a second section, where we will reproduce the results of the empirical application in the main text. Throughout this text, we assume that the R packages `mirt` and `strucchange` have been installed and loaded. After their installation, these packages can be loaded via:

```
> library(mirt)
> library(strucchange)
```

1 R Functions for Running the Tests

1.1 The Pooled Variance Approach

This method can be implemented quite easily by building upon the existing R packages `mirt` and `strucchange`. It is still necessary to define a function that centers the individual score contributions. In `strucchange`, these functions are typically named estfun, and we will also use a similar name here to stay consistent. A centering of the score contributions can be achieved via:

```
> estfun_PooledVariance <- function(x){
+   ### Obtain the score contributions from a model estimated by
+   ### the mirt package
+   scores <- mirt::estfun.AllModelClass(x)
+
+   ### Center the score contributions
+   corrector <- apply(scores, 2, mean)
+   scores <- t(apply(scores, 1, function(x) x - corrector))
+
+   return(scores)
+ }
```

1.2 The Simulation-based Approach

The simulation-based approach is more difficult to implement. We have to 1) center the score contributions and carry out the group-wise decorrelation, 2)

simulate stochastic processes that serve as reference models, and 3) calculate a suitable test statistic for all stochastic processes to finally obtain p-values. We start by defining another estfun function for the first step of the calculation, that is, the centering and decorrelation the individual score contributions:

```
> estfun_groupwise <- function(x){
+   ### Obtain the score contributions from a model estimated by
+   ### the mirt package
+   scores <- mirt::estfun.AllModelClass(x)
+
+   ### Center the individual score contributions
+   corrector <- apply(scores, 2, mean)
+   scores <- t(apply(scores, 1, function(x) x - corrector))
+
+   ### Decorrelate the score contributions
+   # Get the groups from the mirt object
+   group <- extract.mirt(x, what = "group")
+
+   # Get the number of items
+   i <- ncol(extract.mirt(x, what = "data"))
+
+   # Create a matrix for the decorrelated scores -
+   # since the score contributions obtained from mirt contain
+   # two columns for score contributions related to the group
+   # parameters, we have to subtract two columns if we consider
+   # two groups and model the mean and variance in the 2nd group.
+   newscores <- matrix(nrow = nrow(scores), ncol = ncol(scores)-2)
+
+   # Repeat for each group
+   for (a in levels(group)) {
+     # Obtain the group-wise score contributions
+     subset <- scores[group == a,1:ncol(newscores)]
+
+     # Obtain the number of respondents in this group
+     n <- nrow(subset)
+
+     # Decorrelate the score contributions, based on code from strucchange
+     subset <- subset/sqrt(n)
+     J <- crossprod(subset)
+
+     J12 <- root.matrix(J)
+
+     subset <- t(chol2inv(chol(J12)) %*% t(subset))
+
+     subset <- subset * sqrt(n)
+
```

```
+     newscores[group == a,1:ncol(newscores)] <- subset
+   }
+
+   return(newscores)
+ }
```

We still need a function for the remaining steps of the calculation. A suitable function for the unordered Lagrange Multiplier statistic and a categorical covariate, saved by a factor, is provided by the following function. Please see the main text for a definition of this statistic.

```
> LMuo_p <- function(score, covariate, sims = 1000){
+
+   # Function for calculating the test statistic using
+   # a matrix of individual score contributions and a categorical covariate
+   LMuo <- function(scores = score, covariates = covariate){
+     # A helper matrix that includes the sums of score contributions ordered
+     # for each category of the covariate
+     helper <- matrix(nrow = length(levels(covariates)), ncol = ncol(scores))
+     for (a in levels(covariates)) {
+       helper[which(levels(covariates) == a),] <- apply(scores[covariates == a,]
+                                                        , 2, sum)
+     }
+     # Calculation of the test statistic
+     LMuo <- 0
+     for (a in 1:nrow(helper)) {
+       if (a == 1) {
+         LMuo <- sum(helper[a,]^2)
+       } else {
+         LMuo <- LMuo + sum((helper[a,] - helper[a-1,])^2)
+       }
+     }
+     return(LMuo)
+   }
+
+   # Calculation of the test statistic for the observed process
+   LM_obs <- LMuo()
+
+   # We define a vector to store the reference distribution
+   LMuo_ref <- vector(length = sims)
+
+   # The column-wise mean of scores for calibration
+   mean_scores <- apply(score, 2, mean)
+
+   # Repeat sims times
+   for (a in 1:sims) {
+
```

```
+     # Create a matrix for an artificial stochastic process
+     scores_sims <- matrix(nrow=nrow(score), ncol = ncol(score))
+
+     # For each item parameter, draw from an univariate normal distribution
+     # to mimic a multivariate normal distribution
+     for(b in 1:ncol(scores_sims)){
+       scores_sims[,b] <- rnorm(n = nrow(scores_sims))
+     }
+
+     # Carry out a group-wise decorrelation
+     newscores <- matrix(nrow = nrow(scores_sims), ncol = ncol(scores_sims))
+
+     for (b in levels(covariate)) {
+       subset <- scores_sims[covariate == b,1:ncol(newscores)]
+
+       n <- nrow(subset)
+
+       subset <- subset/sqrt(n)
+       J <- crossprod(subset)
+
+       J12 <- root.matrix(J)
+
+       subset <- t(chol2inv(chol(J12)) %*% t(subset))
+
+       subset <- subset * sqrt(n) ### Leads to unity matrix
+
+       newscores[covariate == b,1:ncol(newscores)] <- subset
+     }
+     scores_sims <- newscores
+
+     # Adapt the mean so that it matches that of the
+     # observed stochastic process
+     for(b in 1:ncol(scores_sims)){
+       scores_sims[,b] <- scores_sims[,b] - mean(scores_sims[,b]) + mean_scores[b]
+     }
+
+     # Calculate the test statistic and store it
+     LMuo_ref[a] <- LMuo(scores = scores_sims, covariates = covariate)
+   }
+
+   # Calculate and return the p-value
+   return(mean(LM_obs < LMuo_ref))
+ }
```

2 An Application with Empirical Data

In this section, we demonstrate the application of the functions presented in the first section using the MathExam14W dataset from the `psychotools` package. We start by loading the dataset:

```
> library(psychotools)
> data("MathExam14W")
```

For convenience, we store the responses and the group covariate as separate objects:

```
> resp <- MathExam14W$solved
> group <- MathExam14W$group
```

Next, we want to define our estimation method. We want to compare a) an MML estimator and b) an MAP estimator with flat prior distributions for the slope, intercept and pseudo-guessing parameters. In `mirt`, both can be done by defining suitable models by a special syntax. More information is provided in the documentation of `mirt`. The model for the MML estimator is simply:

```
> model_ML <- 'F = 1-13'
```

In nontechnical terms, this syntax essentially states that items 1-13 are intended to measure a single latent trait. For the MAP estimator, we want to use a N(1,10) prior for the slope parameters, a N(0,10) prior for the intercept parameters, and a B(1,1) prior, which is an uniform distribution, for the pseudo-guessing parameters. This is defined via:

```
> model_MAP <- 'F = 1-13
+       PRIOR = (1-13, g, expbeta, 1, 1),
+               (1-13, a1, norm, 1, 10),
+               (1-13, d, norm, 0, 10)'
```

In our test, we want to check the hypothesis that the item parameters are invariant accross both groups. First, we define `constr`, which allows us to constrain all item parameters to be invariant for both groups (for more details, see the documentation of the `mirt` package):

```
> n_params <- ncol(resp)*4
> constr <- c(lapply(seq(1, n_params, 4), function(x) c(x, x + n_params + 2)),
+             lapply(seq(2, n_params, 4), function(x) c(x, x + n_params + 2)),
+             lapply(seq(3, n_params, 4), function(x) c(x, x + n_params + 2)))
```

We are then able to estimate the parameters using both methods and to carry out the tests. The item parameter estimation is done via:

```
> res_ML <- multipleGroup(data=resp, model=model_ML, itemtype='3PL',
+                         group = factor(group),
+                         invariance = c("free_means", "free_var"),
```

```
+                              constrain = constr,
+                              TOL = 1e-7, technical = list(NCYCLES = 50000))
> res_MAP <- multipleGroup(data=resp, model=model_MAP, itemtype='3PL',
+                               group = factor(group),
+                               invariance = c("free_means", "free_var"),
+                               constrain = constr,
+                               TOL = 1e-7, technical = list(NCYCLES = 50000))
```

This code estimates a multiple group IRT model that assumes a normal distribution of the person parameters in each group, but allows their means and variances to differ in each group. The TOL argument sets the threshold for a convergence of the EM algorithm, which underlies the estimation algorithm, to 1e-7, whereas the NCYCLES arguments sets the maximum number of iterations of the EM algorithm to 50000.

We can now apply the pooled variance approach via:

```
> sctest(res_ML, order.by = group,
+        parm = seq(1, ncol(resp)*3),
+        scores = estfun_PooledVariance, functional = "LMuo")$p.value
> sctest(res_MAP, order.by = group,
+        parm = seq(1, ncol(resp)*3),
+        scores = estfun_PooledVariance, functional = "LMuo")$p.value
```

The simulation-based approach is applied via:

```
> scores_ML <- estfun_groupwise(res_ML)
> scores_MAP <- estfun_groupwise(res_MAP)
> LMuo_p(score = scores_ML, covariate = factor(group))
> LMuo_p(score = scores_MAP, covariate = factor(group))
```

We get p-values close to 0, which indicates a violation of the tested null hypotheses, and thus a violation of parameter invariance.
